# Placebo Analgesia Affects Brain Correlates of Error Processing

**DOI:** 10.1371/journal.pone.0049784

**Published:** 2012-11-21

**Authors:** Leonie Koban, Marcel Brass, Margaret T. Lynn, Gilles Pourtois

**Affiliations:** 1 Laboratory for Neurology and Imaging of Cognition, Department of Neurosciences, University of Geneva, Switzerland; 2 Swiss Center for Affective Sciences, University of Geneva, Geneva, Switzerland; 3 Department of Experimental Psychology, Ghent University, Ghent, Belgium; 4 Department of Experimental Clinical and Health Psychology, Ghent University, Ghent, Belgium; University of Manchester, United Kingdom

## Abstract

Placebo analgesia (PA) is accompanied by decreased activity in pain-related brain regions, but also by greater prefrontal cortex (PFC) activation, which has been suggested to reflect increases in top-down cognitive control and regulation of pain. Here we test whether PA is associated with altered prefrontal monitoring functions that could adjust nociceptive processing to a mismatch between expected and experienced pain. We recorded event-related potentials to response errors in a go/nogo task during placebo vs. a matched control condition. Error commission was associated with two well-described components, the error-related negativity (ERN) and the error positivity (Pe). Results show that the Pe, but not the ERN, was amplified during placebo analgesia compared to the control condition, with neural sources in the lateral and medial PFC. This Pe increase was driven by participants showing a placebo-induced change in pain tolerance, but was absent in the group of non-responders. Our results shed new light on the possible functional mechanisms underlying PA, suggesting a placebo-induced transient change in prefrontal error monitoring and control functions.

## Introduction

Placebo analgesia (PA) refers to a reduced pain sensation due to the belief in an otherwise non-effective treatment [1], [2]. During the last decade, advances have been made in understanding the neurophysiological mechanisms and psychological processes underlying the generation and maintenance of PA. Converging neuroimaging evidence demonstrates that placebo effects are accompanied by decreases of pain-related brain activation in the so-called ‘pain matrix’ [3], [4], [5], [6]. Such decreases are consistently paralleled by *increased* activity in other regions, such as lateral and medial prefrontal cortex (LPFC and MPFC) – both during PA [3], [4], [7], [8] and as early as during expectation of relief, before actual pain stimulation [4], [6]. However, thus far there is no conclusive evidence regarding the *functional* role of these prefrontal areas in PA [9]. Several authors have suggested that LPFC activations during PA reflect a recruitment of cognitive control mechanisms that could in turn trigger opioidergic changes in the descending pain inhibitory system (e.g. [6], [9], [10], [11], [12]). Here we address the question of whether PA could be intertwined with a key function of prefrontal cognitive control networks, namely error processing.

In line with this hypothesis, previous research has shown that the expectation of pain relief is crucial for the implementation of the analgesic response [10]. In their seminal study, Wager et al. [6] reported enhanced activations in LPFC and MPFC, along with orbitofrontal regions during the anticipation of immediate pain administration under PA. Remarkably, brain activity in these cognitive control regions, including LPFC, MPFC, and parietal regions, predicted inter-individual differences in placebo responses [13]. Further, transient and reversible inhibition of LPFC by means of repetitive transcranial magnetic stimulation (rTMS) was found to prevent PA, suggesting a causal role of this cognitive control region for the implementation of placebo analgesia [12].

**Figure 1:**
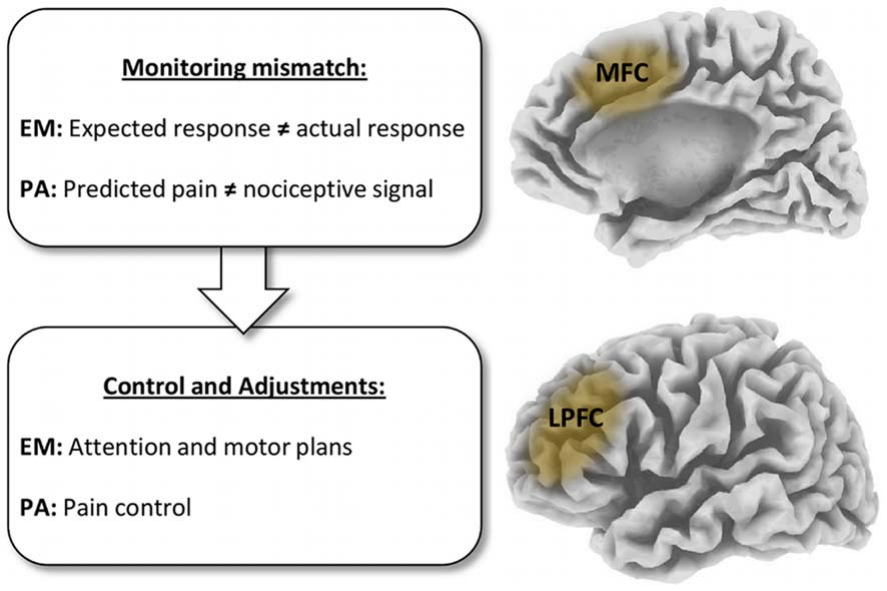
Conceptualizing placebo analgesia (PA) and error monitoring (EM) in a common cognitive control framework. Both EM and PA require the detection of conflicts or prediction errors, and subsequent adjustments in cognitive processing. Thus, they could be based on similar prefrontal functional mechanisms and interact with each other.

We reasoned that the functional role of prefrontal cognitive control regions such as LPFC and MPFC during maintenance of PA may be related to the monitoring and the regulation of upcoming nociceptive input. During PA, the brain has to adjust to a mismatch between predicted pain and actual nociceptive input (i.e. a prediction error). PA may thus require two complementary processes, which are also crucial for error monitoring, and central to cognitive control in general [14], [15], [16]: first, PA probably requires *monitoring* of prediction errors between expected and actual nociceptive signals, and second, subsequent adjustments in top-down *control* of neural processing (see Fig. 1). Strikingly, the specific brain regions showing enhanced activation during placebo analgesia, most notably medial and lateral PFC areas, were previously associated with these particular functions (e.g. [17]). Several influential theoretical accounts of cognitive control converge in their proposition that MPFC is crucial for the monitoring of conflicts, prediction errors, and other negative or at least surprising events that require adjustments in cognitive control [14], [17], [18], [19], [20], [21], [22]. The necessary subsequent adjustments in top-down control are thought to be implemented in more lateral prefrontal regions [14], [17]. In light of this model, we reasoned that if the implementation of PA depends on the same prefrontal brain mechanisms as more general control processes, then PA may in turn also exert influences on error processing brain processes (see Fig. 1).

Previous electrophysiological studies have identified event-related potentials (ERPs) specifically associated with early error detection. Response errors typically induce a large negative ERP component (termed the error-related negativity, or ERN) peaking within the first 100 ms following response onset over frontocentral scalp electrode positions [23], [24]. It is commonly assumed that the ERN reflects an automatic conflict or prediction error signal generated in the MPFC [19], [23], [25], [26]. The ERN is followed by the error positivity (Pe), a large positive deflection peaking between 100–300 ms with a more central scalp topography [27], [28], [29], [30]. The Pe appears to be generated by similar MPFC regions as the ERN, as well as additional sources, including the LPFC, insula, and orbitofrontal cortex [31], [32]. ERN and Pe are often found to be functionally dissociated [28]. Whereas the ERN is thought to reflect a generic error/conflict detection process based on internal motor representations [14], [19], [33], the functional significance of the Pe is related to more elaborate stages of error processing, including subsequent adjustments in behavioral control and awareness of error commission [27], [28], [33], [34], [35], [36].

If the assumption holds true that PA is based on cognitive mechanisms that functionally overlap with cognitive control processes related to conflict monitoring, one would expect PA to have a direct influence on such electrophysiological markers of error processing. The goal of our study was to test whether PA may be associated with specific changes in the aforementioned monitoring processes. On the one hand, one may hypothesize that PA may *decrease* error-related brain responses, as they are generated by mediofrontal mechanisms that may also be sensitive to negative affect, pain, and –more generally– need for control [21]. Yet, it has been shown that the effects of PA are usually quite selective regarding the specific instructions about the treatment effects. For example, only the hand upon which “analgesic” cream is applied showed reduced pain sensitivity and was associated with reduced ACC activity, but not so for the opposing hand [6]. Therefore, it appears unlikely that PA reduces negative affect in general – unless participants were directly instructed that this is what the treatment does (see e.g. [37]).

Alternatively, we predict that PA *amplifies* error-related brain potentials during the expectation of analgesic effects. This hypothesis is based on accumulating evidence showing that *increased* activity in medial and lateral prefrontal areas, which are crucial for cognitive control and regulation of emotion, is predictive for the installation of analgesic effects [6], [9], [10], [12], [13].

Participants were tested using a randomized cross-over within-subject design. They were led to believe that a single (inert) dose of a capsule ingested at the beginning of the experiment was either a reliable painkiller (placebo condition) or an ineffective pill (control condition). Pain threshold and tolerance were measured at the beginning and at the end of each experimental session to quantify the individual placebo response to a standard thermal pain administration on the forearm. During the experimental session, 64-channel EEG was recorded while participants performed a speeded go/nogo task and occasionally committed unwanted response errors (i.e. false alarms on nogo stimuli). This enabled us to characterize error-related brain activities during PA versus a perfectly matched control condition, using a within-subject design. Based on the hypothesis that common brain areas underlie both PA and error monitoring (see Fig. 1), we predicted that PA would have an amplifying effect on these ERP components specifically related to error detection.

## Materials and Methods

### Participants

Twenty healthy undergraduates from Ghent University (14 women; all right-handed as determined by self-report; mean age 21.2 years; range 18–31) participated in two experimental sessions (placebo and control), both taking place at the same time on two consecutive days. Session order was counterbalanced across participants. In order to enhance the plausibility of the cover story, the experimenter was wearing a white medical coat and conducted a brief interview at the beginning of the first session, in which participants were thoroughly screened for any history of serious physical, mental, or neurological illness, for pain-related disorders, allergies, and current medication. Two female participants had to be excluded from the analysis, one because she did not believe in the cover story used to induce PA, and another for reporting that she did not experience any temperature increase during thermal pain stimulation. Accordingly, the final sample contained 18 naïve participants. The study was conducted in accordance with the Declaration of Helsinki and approved by the ethics committee of the Faculty of Psychological and Educational Sciences, Ghent University. All participants gave informed written consent, and were compensated 40€.

### Stimuli and Task

The speeded go/nogo task used in our study has been extensively described elsewhere [29], [30], [32], [38], [39]. Each trial started with a central fixation cross (presented for 1 s) followed by a black arrow that changed color after a randomly jittered interval of 1–2 s. In two thirds of the trials (go-trials), the arrow turned green, indicating that participants should respond as quickly as possible by pressing the space bar. In the remaining third of trials (nogo), the arrow turned either cyan (instead of green) or turned green, but changed direction (relative to the black arrow), indicating participants had to withhold their response. One second after the response, feedback was given in the form of an isoluminant green or red dot for correct versus incorrect responses, respectively. In order to increase commission errors, only fast responses, as determined by calibrating an individual RT limit updated on a trial-by-trial basis [29], [30] were classified as correct. This imposed speed pressure promoted the occurrence of many responses errors, allowing for reliable error-related ERP waveforms in each condition. Each of the six blocks consisted of 60 trials (40 go and 20 nogo), resulting in 360 trials in total (∼30 min).

### Apparatus

Painful thermal stimulation and threshold tests were administered using a MSA Thermotest device (SOMEDIC Sales AB, Sweden), with the thermode placed on the left or right volar forearm, and controlled using the manufacturer’s software. The experiment took place in an electromagnetically shielded, dimly lit chamber, with participants seated 80 cm in front of a computer screen. Stimulus presentation was controlled using E-Prime 2.0 software.

### Procedure and Measures

#### Placebo induction

Volunteers were told they were taking part in a study investigating the effects of a widely used painkiller on EEG activity during an “attention task.” Prior to the experiment, participants read an information sheet about the medication, its analgesic properties (“highly effective in reducing pain on many body parts, including heat pain”), the onset (“about 11 minutes after oral administration”), and duration (“2–4 hours”) of these effects. Following the first pain threshold measurement, participants were given a capsule containing 160 mg of Mannitol (a medically non-effective white sugar substitute) and a glass of water. They were given instructions aimed at inducing either an analgesic placebo or a neutral effect: “This is a capsule of an effective pain reliever. In 10–15 minutes the medication will be fully effective, and notably decrease your sensitivity to the thermal heat pain” (Placebo condition) or “This is a capsule without any effective drug, needed as a control. It will not decrease your sensation of pain nor induce any other effects” (Control condition).

#### Measurement of pain sensitivity

**Figure 2:**
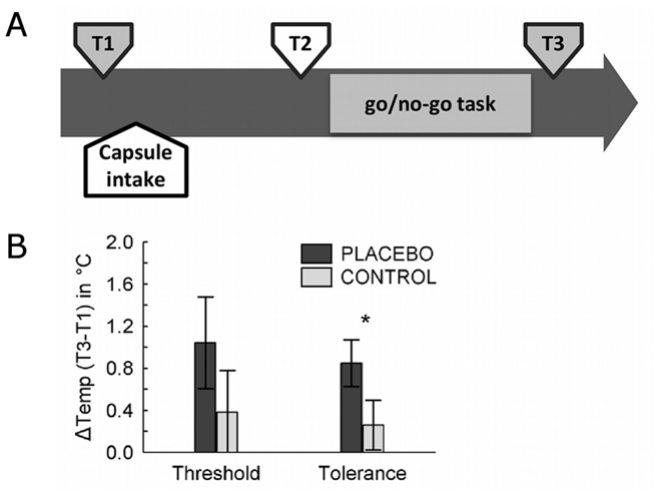
Experimental design. Measures of pain sensitivity were obtained at baseline just prior to placebo capsule administration (T1), 15 min after capsule intake in order to enhance placebo beliefs (T2), and at the end of the experiment, following the go/no-go task (T3), in order to measure the individual placebo response (A). Average change in pain sensitivity, as measured by pain threshold and pain tolerance increases, comparing T3 to T1 baseline (B). Vertical bars denote standard errors.

Pain tolerances and thresholds were determined at three intervals during each session (see Fig. 2A): Prior to intake of the Mannitol capsule (T1, corresponding to the baseline); after installing the EEG cap and electrodes (∼20 minutes after intake), in order to establish the placebo belief (T2); and directly after the go/nogo task (T3, as a manipulation check). Thresholds were assessed by applying a steadily increasing thermal stimulation (starting at 32°C, with a slope of 2°C/s) to the inner wrist. On four consecutive trials (with 5 s breaks between trials), participants were instructed to press a button in order to cease the thermal stimulation, thereby terminating the trial, at the moment the sensation changed from that of heat to pain (pain threshold measure). In a further four trials, participants were requested to button press to indicate the point at which the pain became unbearable (pain tolerance measure). For each time point, both pain threshold and pain tolerance were calculated as the average temperature across the four trials. The net placebo effect upon threshold and tolerance was quantified as the difference between the differences (interaction term) of the temperatures in T3 versus T1 between placebo and control condition (T1_Placebo_–T3_ Placebo_)–(T1_ Control_–T3_ Control_). Participants did not receive feedback as to temperature values. However, in order to enhance the placebo induction, following the second threshold measurements (T2) participants were told (irrespective of the actual values): “Both your pain threshold and your pain tolerance have significantly increased.” (Placebo condition) or “Your threshold are very similar to the ones obtained in the first test.” (Control condition).

Further, two single-pulse thermal stimulations, lasting 5 s, were administered at three time points for each session. Participants were informed that this stimulation served to determine subjective changes in pain intensity throughout the experiment, and were asked to rate the pain intensity of the pulse on a scale of 0–8, with 0 being no pain, and 8 being the greatest pain imaginable. The single-pulse stimulations were administered directly after the threshold and tolerance measurements for T1, but directly before measurement for T2 and T3. In all cases but one, the pulse stimulation was identical to the T1 threshold and tolerance averages; unbeknownst to the participants, we lowered the single-pulse threshold and tolerance temperatures by 1.5°C in the placebo condition at T2 (immediately before the go/nogo task) in order to mimic a genuine analgesic effect of the administered pill [6].

In order to assess belief in the placebo manipulation, participants indicated their expectancy of the analgesic effect of the pill they ingested following each experimental session in a questionnaire. They responded to the item “Did you expect an effect of the capsule intake on your pain sensitivity?” on a Likert-scale from 1 to 7 (Analgesia expectancy). To check for the presence of a subjectively experienced analgesic effect, we asked: “How efficient was the capsule in reducing the pain sensation when you received thermal pain?” After the second experimental session, all participants were thoroughly debriefed and screened for possible suspicion concerning the credibility of the cover story.

### Additional Behavioral Measures

To control whether potential effects were mediated by mood changes or by other beliefs participants might have, we measured state anxiety at the end of each EEG session using the validated Dutch version of the STAI [40], and included two items at the end of the second session concerning the expectancy of medication-related cognitive performance increase and decrease (“Did you expect an improvement of your performance in the attention task, following the intake of the pain medication capsule?” and “Did you expect a decrease in your performance in the attention task, following the intake of the pain medication capsule?”).

### EEG Preprocessing and Analyses

EEG was recorded from 64 electrodes with a sampling rate of 2048 Hz (ActiveTwo Biosemi system). Following standard practice, raw data were re-referenced offline to a common average and filtered (0.5–30 Hz, and 50 Hz notch). Following ocular correction using an ICA algorithm as implemented in Brain Vision Analyzer 2.0 software, the data was segmented from −500 to 1000 ms around responses, separately for fast correct hits and commission errors. Segments exceeding amplitudes of ±80 µV were excluded prior to baseline correction and averaging. Individual average ERP waveforms were filtered using 1–30 Hz.

In order to assess the time windows of error-related components in a unbiased and objective, data-driven way, we first calculated the grand average ERP waveform for errors across both placebo and control condition and identified the ERN and Pe components based on two reference-free global measures of the electric field, dissimilarity and global field power (GFP) [41]. GFP measures the overall “energy” of the electrical field on the scalp, by summing up the squared field potentials across all electrodes. Conversely, dissimilarity measures the change in the topographical distribution (irrespective of changes in the local or global strength of the ERP signal), and is therefore highly sensitive to transitions between different microstates or ERP components [41], [42], [43], [44]. ERN and Pe were identified as phases of high global field power, between local maxima of topographical dissimilarity [41], [42], [43], [44]. Thus, we extracted the ERN amplitude as the minimum value between −40 and 60 ms around response, and the Pe as the maximum between 100–280 ms, for errors and fast hits separately in the two experimental conditions (placebo versus control), at the electrode sites Fz, FCz, and Cz, where these two error-related components were found to be maximal. Slow hits were not further analyzed, as they are characterized by different RT distributions compared to fast hits or errors [29], [30].

### Source Analysis

We used standardized low resolution brain electromagnetic tomography (sLORETA) [45] to estimate the neural generators underlying the increased Pe amplitude in the placebo condition (see results below). In order to deal with the inverse solution problem and restrict the number of possible solutions, sLORETA assumes maximal “smoothness” of the current density, and further restricts the possible three-dimensional solutions to 6239 points in the cortical grey matter volume. With a regularization parameter of SNR  = 10, source activity was first estimated for the individual ERPs, separately for fast hits and error trials in the placebo versus the control condition.

### Statistical Analyses

Behavioral and ERP data were analyzed by means of repeated-measures ANOVAs or mixed-effects ANOVAs for group comparisons (placebo responders versus non-responders). Planned pairwise t-tests were Bonferroni-corrected for multiple comparisons. Statistically significant differences in the source space (sLORETA analysis) were evidenced using paired t-tests (uncorrected for multiple comparisons, given the smoothness of source activity estimation) performed on the mean activity from 100 to 280 ms after response onset (encompassing the Pe component).

## Results

### Behavioral

#### Manipulation check

Questionnaire results revealed a strong effect of placebo versus control condition on analgesia expectation (Placebo mean = 4.61, SD = 1.69, Control mean = 1.33, SD = 0.77; *t*(17) = 8.1, *p*<0.001) and on subjectively experienced pain relief (Placebo mean 4.44, SD = 1.04, Control mean = 1.44, SD = 0.51; *t*(17) = 13.1, *p*<0.001), confirming that participants experienced a subjective pain-relieving effect of the capsule in the placebo compared to the control session.

Changes in subjective pain intensity ratings (Likert scale 0–8) for the threshold temperature were tested using a repeated-measures ANOVA with the factors TIME (baseline T1 vs. T3) and CONDITION (placebo vs. control). We found a significant main effect for TIME (*F*(1,17) = 8.6, *p* = 0.008), but no significant main effect for CONDITION, and no significant interaction. The same statistical analysis run on the ratings obtained for the tolerance temperature revealed no main effect of CONDITION, but trends for the main effect of TIME (*F*(1,17) = 4.1, *p* = 0.059), as well as for the TIME×CONDITION interaction (*F*(1,17) = 4.1, *p* = 0.058). This latter interaction was driven by a significant increase in pain intensity ratings at T3 compared to T1 in the control condition (Bonferroni-corrected t-test, *p* = 0.011), which was not present for the placebo condition (*p* = 1.00). The direction of the interaction effect was thus in accord with the prediction of analgesic effects following placebo beliefs.

Further, we tested whether effects of PA could be supported in direct and more implicit measures of pain sensitivity, namely via changes in pain threshold and pain tolerance temperatures (see Fig. 2B). Whereas a main effect for TIME was found for both pain threshold (*F*(1,17) = 9.2, *p* = 0.026) and pain tolerance temperature (*F*(1,17) = 10.1, *p* = 0.006), the interaction CONDITION×TIME yielded a trend only for the pain tolerance (*F*(1,17) = 4.0, *p* = 0.061), with no significant effect for the threshold (*F*(1,17) = 1.3, *p* = 0.28). Planned comparisons with Bonferroni-corrected t-tests confirmed that the difference between T3 and T1 (baseline) pain tolerance was significant only in the placebo condition (*p* = 0.005), but not in the control condition (*p* = 1.0), in line with our hypothesis. We note that, in accordance with previous studies on placebo effects [13], our sample contained substantial inter-individual variability regarding placebo responsiveness. We found that only 10 out of 18 participants actually showed a differential increase of pain tolerance temperature (>0.5°C) in the placebo compared to the control condition.

#### Performance during the go/nogo task and other measures

The distribution of, and mean RTs for errors, correct fast hits, and correct slow hits did not significantly differ between placebo and control conditions (see Table 1), indicating a very similar performance across conditions. We did not observe a significant difference in levels of state anxiety between the two sessions, as measured with the STAI (placebo 40.8 versus control 42.1, *t*(17) = 1.3, *p* = 0.22).

**Table 1:** Behavioral results. Means and standard deviations (SD) of trial numbers and RTs for the different response conditions in the two experimental sessions (Placebo versus Control).

	Number of trials−Mean (+1SD)		Reaction times−Mean (+1SD)	
	Placebo	Control	Placebo	Control
Fast Hits	82.1 (18.4)	83.1 (16.1)	232.1 (30.2)	234.6 (22.7)
Slow Hits	155.8 (18.8)	153.8 (16.6)	315.3 (22.9)	322.9 (21.8)
Errors	46.1 (21.3)	49.2 (18.7)	250.4 (30.0)	255.7 (22.2)

None of these mean numbers differed significantly between conditions.

### Error-Related ERPs

**Figure 3:**
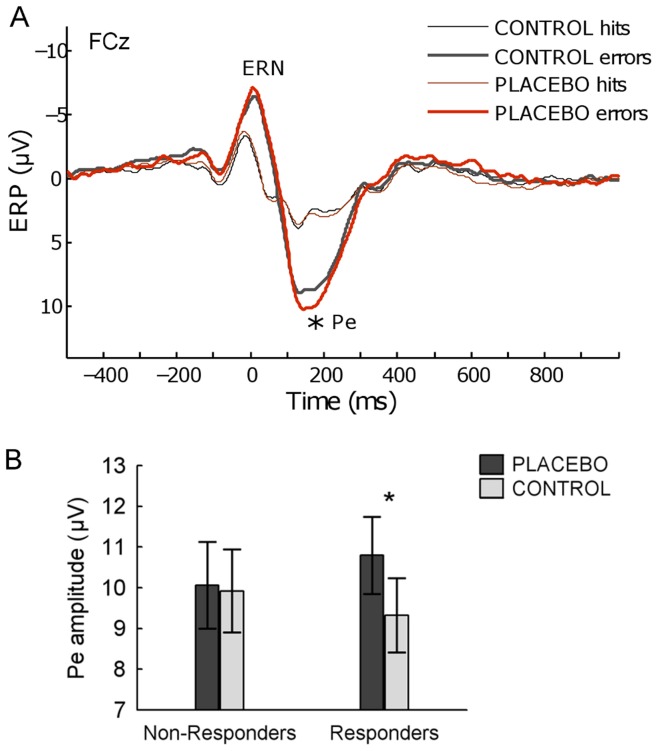
Grand average ERPs for errors and correct hits in placebo and control conditions at electrode FCz. (A). Pe amplitudes (mean across Fz, FCz, and Cz) for placebo responders and non-responders separately, for placebo and control sessions. Only placebo-responders showed an increased Pe response to errors in the placebo condition (B). Vertical bars denote standard errors.

Grand average event-related waveforms are depicted in Figure 3A. A repeated measures ANOVA including the within-subject factors RESPONSE (errors versus correct hits) and CONDITION (placebo versus control), carried out on the ERN amplitudes averaged across the three frontocentral electrodes (Fz, FCz, and Cz), revealed a highly significant main effect of RESPONSE (*F*(1,17) = 25.0, *p*<0.001), showing, as expected, a larger ERN for errors relative to fast hits. However, the interaction term was not significant *F*(1,17) = 1.6, *p* = 0.23), suggesting that this early error-related activity was not significantly influenced by PA. Further, the ERN did not significantly correlate with changes in pain tolerance (r = 0.11, p = 0.66), and was not different between placebo responders versus non-responders (GROUP×RESPONSE×CONDITION interaction, *F*(1,16) = 1.9, *p* = 0.18).

A different statistical outcome was found for the Pe component. As expected, the mean Pe amplitude was also significantly larger for errors than correct hits (*F*(1,17) = 44.9, *p*<0.001), but critically, this error-specific component was also modulated by PA, as evidenced by a significant RESPONSE×CONDITION interaction (*F*(1,17) = 7.0, *p* = 0.017). Planned comparisons using Bonferroni corrected t-tests showed that Pe amplitudes were larger for errors under placebo compared to control condition (*p* = 0.004), whereas no difference was seen for amplitudes in response to fast hits (*p* = 1.0), confirming that PA influenced error monitoring selectively.

To further investigate whether this enhanced Pe amplitude was related to inter-individual differences in the placebo response, we compared Pe amplitudes between the two sessions, separately for the placebo-responders versus non-responders (see Figure 3B). The interaction effect between CONDITION and GROUP was significant (*F*(1,16) = 4.7, *p* = 0.045), demonstrating that the Pe amplitude significantly increased for the placebo condition in the responders (Bonferroni-corrected t-test, *p* = 0.014), but not in the non-responders (*p* = 1.0). Further, individual differences in placebo response (tolerance increase) correlated positively with the Pe-amplitude effect (Spearman’s *Rho* = 0.46, *p* = 0.055).

### Source Reconstructions

**Figure 4:**
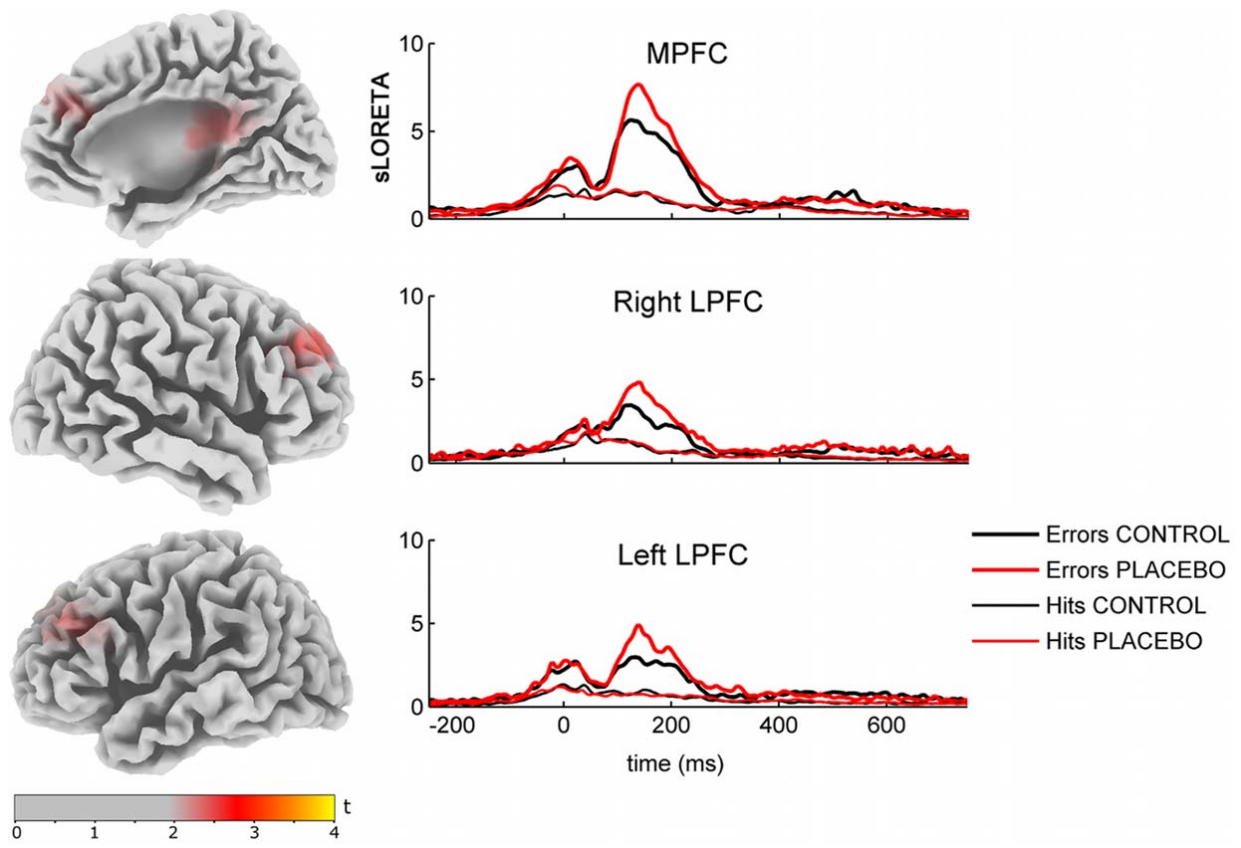
sLORETA source estimations for the statistical interaction effect [PLACEBO ERRORS>PLACEBO HITS]>[CONTROL ERRORS>CONTROL HITS] on the Pe component, 100–280 ms after response (p<0.05 uncorr.). (A). Time courses of the three ROIs for errors and hits in both placebo and control conditions (B).

In order to gain insight into the brain generators underlying the modulation of the Pe component with PA, we used sLORETA to investigate which brain regions showed a significant interaction effect between RESPONSE×CONDITION around the Pe component (100–280 ms). This statistical source analysis revealed three clusters with higher error-specific activity during the placebo condition, localized in the MPFC (MNI peak coordinates x = 20, y = 35, z = 30), left LPFC (x = −15, y = 50, z = 45), and right LPFC (x = 45, y = 30, z = 25, see Table 2 for detailed results). In each of these three regions, we found significantly higher activation for the placebo than the control condition, specifically for error trials (see Figure 4).

**Table 2:** Results of the sLORETA source estimation for the contrast [Placebo Errors>Placebo Hits]>[Control Errors>Control Hits] during the time window of the scalp Pe effect, 100–280 ms following the response.

Region	Lat	Max *t*-value	MNI-coordinates			Number of voxels
			x	y	z	
Cingulate gyrus	R/L	2.10	15	25	40	4
Medial frontal gyrus	R/L	2.33	20	35	30	47
Inferior frontal gyrus	R	2.48	50	25	25	7
Middle frontal gyrus	R	2.78	45	30	25	66
Precentral gyrus	R	2.33	35	−5	40	25
Postcentral gyrus	R	2.15	40	−25	40	3
Subgyral	R	2.23	35	−5	45	4
Superior frontal gyrus	R	2.69	40	35	35	37
Middle frontal gyrus	L	2.11	−30	35	35	12
Superior frontal gyrus	L	2.28	−15	50	45	16
Precuneus	L	2.09	−15	−60	25	3

All activations are thresholded at *p*<0.05 (uncorrected).

## Discussion

Placebo effects are a compelling example of how perception may be profoundly shaped by expectations and social instructions, and more specifically how the generation and maintenance of beliefs in a pain-relieving treatment may regulate painful experiences. Despite a growing number of studies looking at the neural and pharmacological mechanisms, as well as psychological processes underlying this fascinating effect, little is known about the potential functional contribution of prefrontal cognitive control areas during PA. Previous studies have demonstrated that prefrontal areas (especially DLPFC and MPFC) are crucial for the installation of the analgesic response [3], [12], [13], but so far their functional role in PA remains unclear (cf. [9]). In order to test the assumption that PA could involve a recruitment of mechanisms similar to those employed in error monitoring, we compared error-related ERPs during PA versus a properly matched control condition, using a cross-over within-subject design.

Our results demonstrate for the first time that PA is related to altered error monitoring brain processes at the level of the Pe component. Importantly, additional analyses confirmed that this PA-dependent effect upon the Pe was primarily driven by those participants who actually showed a placebo effect (increased pain tolerance), providing additional evidence that the Pe increase was linked to the analgesic effect of our manipulation. Remarkably, source reconstructions obtained using sLORETA confirmed that this effect was likely caused by increased activation of specific medial frontal as well as lateral prefrontal regions, which have classically been associated with adaptive control brain mechanisms [14], [17] and more recently with adjustments to social norms and expectations [46], [47], [48], [49], but have also been demonstrated to be crucial for PA [3], [6], [12], [13].

Further, the ERP results suggest that the effects of PA are error-specific. Whereas the processing of correct actions (i.e. fast hits) was not influenced, neurophysiological responses to response errors were modulated by PA. Moreover, only the Pe component, and not the preceding ERN component, was reliably enhanced during PA. An additional analysis, in which we directly compared the GFP peaks of these two components (cf. [50]) did not yield a significant interaction effect between component (i.e. ERN versus Pe) and experimental condition. Hence, we cannot exclude the possibility that PA might also influence conflict or error processing at the level of the ERN. Whereas the ERN probably indexes an initial automatic stage of prediction error or motor mismatch detection in dMFC [14], [19], [35], [51], the Pe has been related to later stages of error processing, leading to error awareness and subsequent adaptive changes in behavior [28], [32], [35]. Consistent with this framework, it has been argued that the Pe component reflects context updating, thus swiftly signaling error salience and the need for adjustments in cognitive control [27], [28], [36]. Accordingly, our novel results of a Pe- effect suggest that PA may induce a transient change in the reactivity of cognitive control networks. These networks are probably necessary in order to adjust to a mismatch between predicted and experienced pain (see Fig. 1) [52], to modulate nociception by top-down reappraisal, and to influence opioidergic antinociceptive pathways [6], [11], [12], [53]. This transient increase in top-down cognitive control mechanisms could be general enough to modulate the processing of other events requiring enhanced cognitive control and top-down regulatory adjustments, such as response errors. Our findings also indirectly suggest that PA may depend on an altered balance between top-down expectations and bottom-up nociceptive processing. In line with this reasoning, a recent study [54] demonstrated that individual differences in a cognitive style of biases towards prior expectations were related to differences in placebo responsiveness. Furthermore, we also note the tight overlap between the source reconstructions underlying the Pe effect in LPFC and previous fMRI activations found in the exact same regions that actually predicted inter-individual differences in placebo responsiveness [6], [13].

These changes in cognitive control processes during PA may be relatively specific to adjustments following mismatch detection, or alternatively, to events requiring emotion regulation [13]: A recent study combined placebo expectation with a working memory task in order to test directly whether PA interferes with executive attention resources [9]. Results showed additive, not interacting, effects of working memory load and placebo expectation on analgesia, suggesting that executive functions recruited during working memory are probably not directly involved in maintaining PA [9]. Consistent with this study, we did not find any differences in behavioral performance during placebo compared to the control condition. On the other hand, the use of an adaptive response deadline might have prevented strong differences in behavioral indices of performance monitoring across the two conditions. However, this procedure was an important prerequisite in order to obtain a balanced number of errors (and hence ERP averages) between sessions. More generally, it is also conceivable that the PA-dependent Pe increase seen here indicates differences in affective error appraisal [35], [36], rather than adjustments in attention or effort. This interpretation would be in line with the notion that PA is mediated by brain regions involved in the regulation of emotion [13].

Our results, showing a modulation of error monitoring by expectation of pain relief, add to accumulating evidence of the importance of prefrontal control mechanisms for placebo effects. To the best of our knowledge, the present ERP results give a first hint on their functional role: PA may in part depend on domain-general cognitive control mechanisms that subsume error processing, mismatch detection, and subsequent adjustments in cognitive and emotional control (see Fig. 1). Future studies are needed to investigate whether the modulation of adaptive control functions induced by PA could also be observed in emotion regulation or conflict-resolution tasks other than error monitoring, and to characterize the role of individual and social expectations in PA.

Further, more research is needed to establish whether the observed link between error monitoring and placebo analgesia can also be shown in a more general population or alternatively in clinical settings, where pain experience or relief expectations may be altered. Given the relatively low spatial resolution of electrophysiological source estimation and the use of conventional (uncorrected) statistical thresholds in our sLORETA analysis, we also suggest that future studies should employ fMRI (ideally in combination with scalp EEG measurements) in order to investigate the neuroanatomical substrates of PA-mediated effects on error monitoring processes in more detail.

These findings also underscore the malleability of early error and performance monitoring brain processes. They show that these mechanisms are not only influenced by trait-like emotional or motivational factors [39], [55], but also by contextual or state-dependent changes in expectancies or beliefs. Psychosocial contextual factors such as expectation of pain relief may impose powerful modulations upon prefrontal cognitive control networks. As such, our results suggest that beyond environmental, genetic, or neuroanatomical differences, cognitive control and error monitoring brain processes are also readily shaped by intra-individual variations concerning abstract beliefs or social expectations.
